# 

**Published:** 1912-07-06

**Authors:** 


### Books Received.

THE SCIENTIFIC PRESS, LTD.: "Elementary Anatomy and Physiology," by W. Bernard Secretan. Second edition.

CASSELL AND CO., LTD.: "Materia Medica and Therapeutics," by J. Mitchell Bruce and Walter J. Dilling. Ninth edition. 6s. 6d. net.

JOHN MURRAY: "Further Researches into Induced Cell-Reproduction and Cancer," by H. C. Ross. Vol. II. 3s. 6d. net.

OXFORD MEDICAL PUBLICATIONS: "Text-book for Nurses," by Groves and Brickdale.

J. WRIGHT AND SONS: "The New Physiology in Surgical and General Practice," by A. Rendle Short, M.D., B.S. Second edition.

### BUREAU OF INFORMATION.

### Rules for Correspondents.

1. Every letter, on whatever subject, must be accompanied by the coupon to be cut from the back cover (inside page) of The Hospital, current issue, and must contain the name and address of the correspondent with pseudonym for publication if desired.

2. Replies by post cannot be given, save under exceptional circumstances, at the Editor's discretion.

3. Letters from Approved Homes in reply to special needs published in the Bureau should state terms and full particulars, and be sent prepaid under cover to the Editor of the Bureau with coupon and name enclosed for identification.

4. Proprietors of homes which have not yet been entered on the List of Approved Homes, but have spare accommodation likely to suit special needs, are invited to write for an application form for registration. The fee for registration is 5s.

5. Special needs of every description relating to those who are sick or in difficulties are made known in these columns free of charge.

6. All communications to be addressed to The Editor of The Hospital, 28 Southampton Street, Strand, London, W.C., and marked "Bureau of Information."

### Homes Wanted.

### For Retired Army Nurse.

S. M. (112a).—Good home wanted in the South of England for Army nurse permanently in weak health, whose means do not admit of her paying more than 19s. or 20s. a week. She was in the Boer War, but her health broke down before she had served long enough to obtain more than a very slender pension. We think some of our readers may be willing to receive a colleague at these reduced terms, should they have a room to offer, and we will gladly forward letters from the Bureau addressed as above. We think it is a case where a private home would be more congenial than one supported by charitable contributions, but if no suitable offer is received we will make some other suggestion.

### For Mental Patient.

C. N., Normanton (113a).—It is difficult without further particulars to suggest a plan for your son, aged thirty, formerly in the Navy. If you will kindly let us know what terms you are now paying we could better judge what prospect there is of getting him taken at a lesser rate in a private mental hospital. You know, perhaps, that the Holloway Sanatorium, St. Ann's Heath, Virginia Water, receives patients from among the upper and middle classes at very low terms. If you state the circumstances to the secretary, we feel sure they are such as to command consideration. We fear there is no fund which would pay for his maintenance altogether. See also reply under Letter-Box.

### Homes Offered.

### To Nurse for Part Payment.

Yorkshire (10636).—We are very pleased to make it known that you are willing to receive a nurse paying you 5s. a week and giving light services, in return for comfortable home in a healthy district. Since you are a fully-trained nurse living alone with your father, we think you would find it companionable to share your quarters in this way, and the offer may prove very acceptable to North-Country nurses. Prepaid replies forwarded.

### Health Resorts.

### Sark for an Invalid.

Sorella.—We cannot recommend you to take your lame sister to this island, in spite of its beauty and the invigorating nature of the air. The roads are atrocious, driving is a penance, and the distances to the sea from the hotels are quite considerable for bad walkers. None but active persons with a steady head can enjoy Sark as it ought to be enjoyed. Try St. Aubin's, Jersey, where there is a good hotel and easy access to the sea.

### Teneriffe in Summer.

D. B., Liverpool.—The climate is quite tolerable at Orotava even in the hottest weather, owing to the "umbrella" of light cloud from the Peak which mitigates the sun's rays. At the same time, it is somewhat relaxing after April, and residents usually go higher up to Laguna or Tacoronte for a change. There are no English nurses in the Humboldt Hotel, which is now under German management, but a German doctor and German nurse.

### Hospital Appointments.

### Ladies as Secretaries.

Zealous One.—We know of no large general hospital where women are employed in the secretarial department except in the subordinate position of typist, and so on. Men clerks are usually employed, and the work is very similar to that in an ordinary office. In the matron's office women assistants are employed, but these are usually nurses. Some small and special hospitals employ a woman secretary. Study the announcements of vacant posts. Perhaps the post of hospital almoner would suit you, since you are drawn to this kind of work.

### Work Abroad.

### How to Obtain a Post.

Midwife, Sheffield.—Apply to the Colonial Nursing Association, the Imperial Institute, London, S.W. You are mistaken in thinking that this association only sends nurses to the tropics. Since you have three years' general training in addition to the midwifery certificate, you should experience no difficulty in securing the post abroad which you require. There are more vacancies as a rule than satisfactory candidates to fill them.

### A Tempting Offer from Barcelona.

Sister M.—We strongly urge you to consult the British Consul (Mr. J. F. Roberts), Barcelona, Spain, before throwing up your present good post to go out to strangers of whom you know nothing except that they write a plausible letter. You have no security for the payment of your fees, and may find yourself in great straits if these people are unprincipled, especially as you have no knowledge of the language. The climate is delightful.

### Approved Homes.

[Note.—*No Home is added to the List without direct medical testimony to its efficiency.*]

### Nursing Home in Ireland.

Rosebank, Herbert Road, Bray, Co. Wicklow, Ireland.—Principal, Nurse Roberts-Murray, cert. Rotunda Hospital, Dublin. Obstetric, non-infectious medical, slight mental or nerve cases. Sea and mountain air. Established six years. Moderate terms.

### Letter-Box.

Nurse R. M.—We have sent on your letter, and hope your home will prove suitable. Please note that all communications must be accompanied by coupon. If you order The Hospital regularly from your newsagent he will have no difficulty in supplying it.

C. N.—If you would like to register the accommodation you offer, it is very possible that we may have cases which might suit you. We are sending you a form of application that you may see what is required.

Sister E. M.—Very grateful for your kind offer *re* Bureau. Please put it in practice at once.

Bala.—Your query raises an important point, and shall have attention.

*To the Editor of* THE HOSPITAL.

### A CHARING CROSS HOSPITAL SURGEON'S WILL.

SIR FREDERICK CHARLES WALLIS, F.R.C.S., of Harley Street, and formerly of Welbeck Street, W., surgeon to Charing Cross, the Grosvenor, and St. Mark's Hospitals, London, who died on April 26, has left estate of £15,540 gross, and £8,101 net.

### Appointments.

Dr. P. S. Vickerman has resigned his appointment as resident assistant medical officer at Horton Mental Hospital. At Long Grove Mental Hospital, Dr. Bernard Hart, second assistant medical officer, and Dr. H. W. White, fifth assistant medical officer, have resigned their appointments. Dr. Edward Mapother, third assistant medical officer, has been promoted to be second assistant. Mr. Donald A. Macpherson has been appointed junior assistant medical officer.

### Church Rivalry on Hospital Sunday.

The following are among the amounts received at the Mansion House to date: Holy Trinity, Kensington Gore, £154; Holy Trinity, Tulse Hill, £130; St. Mary's, Bryanston Square, £115; St. Stephen's, Gloucester Road, £111; Sir Savile Crossley, £100; Christ Church, Gipsy Hill, £88; St. Andrew's, Leytonstone, £80; Liberal Jews' Synagogue, £74; St. Peter's, Bayswater, £70; St. Paul's, Upper Norwood, £62; Hampstead Parish Church, £58; St. John's, Bromley, Kent, £56; St. Paul's, Beckenham, £53; St. James's, West Hampstead, £51; St. Andrew's, Wells Street, £51; Miss M. E. Druce, £50; Mr. Norman McCorquodale, £50; Farm Street Church, £50; Stamford Hill Congregational Church, £49; Lewisham Parish Church, £48; St. Stephen's, Regent's Park, and Missions, £44; Hayes Parish Church, £41; St. Saviour's, Paddington, £40; Holy Trinity, Upper Tooting, £39; Dulwich College Chapel, £39; St. Paul's, Harringay, £38; Christ Church, Brondesbury, £38; St. John's, Notting Hill, £37; St. James's, Hatcham, £36; St. Mark's, Surbiton, £36; and Bromley Congregational Church, £36.

### Gifts and Bequests.

The Duke of Bedford, President of University College Hospital, has sent a donation of £250 to the General Fund of the Hospital.

Mr. John Gunson, of Ulpha, Cumberland, has left £2,000 to the Ulverston Cottage Hospital, to endow Joseph and Eleanor Gunson beds.

Mr. Bateman Lancaster Rose, of South Kensington, and Scotland, stockbroker, has left £1,000 to the Hospital for Consumption and Diseases of the Chest, Brompton.

An X-Ray Outfit for Hornsey Cottage Hospital.—Mrs. Johnston, of Mount View Road, is presenting a complete *x*-ray outfit for the Hornsey Cottage Hospital, when Messrs. Newton and Wright, who are supplying the apparatus, give an exhibition of radiology.

### The Week's Novelties.

"VIROL" IN CHEST HOSPITALS.

Presiding at the twelfth annual general meeting of Virol, Limited, the Chairman, Mr. B. S. Straus, J.P., said that the year had been one of considerable progress. Their sales had increased, their profits had increased, and this state of things was due to an all-round increase in the home, foreign, and Colonial markets. The business done with hospitals, consumption sanatoria, etc., was (he added) particularly satisfactory. Not only had the hospitals previously using Virol ordered more largely, but the number of such institutions had considerably increased. He believed there was scarcely a hospital, sanatorium, or infirmary of any importance in the country in which Virol was not used and appreciated. Virol had not been adopted in these institutions without examination and scientific test, and they would not have continued to use it if the results had not borne out expectations. He had been greatly struck by the widespread appreciation expressed by medical men as to the value of Virol in the severe epidemic of infantile diarrhœa last summer. He believed that a wider knowledge of the body-building properties of Virol would lead to its still more general use in this connection, and bring about a marked decrease in the present high rate of infant mortality from this and other causes. It was satisfactory also to note the increasing medical recommendation of Virol as a reconstructive agent in adult cases of tuberculosis, and its increasing use in consumption sanatoria.

"CITODA."

Practitioners and institutional superintendents are invited to consider carefully the claims which are put forward by Jewsbury and Brown, of Ardwick Green, Manchester, the manufacturers of mineral waters, on behalf of their special soda-water, "Citoda." Its properties may be indicated by the fact that "sodium citrate in the proportion of five grains to the pint, with an equal quantity of sodium bicarbonate," is among its ingredients. All who are concerned with the problems attending the artificial feeding of infants will recognise sodium citrate as an aid to digestion. There is a certain novelty in applying it to that sick-room classic—soda and milk. It is a refinement of dilution in this respect, which every sick-room worker or nursery or crèche superintendent may be recommended to test practically. It is very pleasant to the palate, and should make milk possess fewer of those disadvantages it may certainly have in an undiluted state.

